# Platelet-rich plasma combined with multimodal therapy for diabetic foot ulcer with tophi: a case report

**DOI:** 10.3389/fcdhc.2026.1795474

**Published:** 2026-04-21

**Authors:** Xinjie Duan, Xinjuan Sun, Yang Liu, Maoyuan Chen, Li Dong

**Affiliations:** 1 Department of Endocrinology, Nanjing Red Cross Hospital, Nanjing, China; 2 Department of Endocrinology, The Air Force Hospital from Eastern Theater of PLA, Nanjing, China

**Keywords:** antibiotic-impregnated bone cement, diabetic foot ulcer, platelet-rich plasma, tophi, traditional Chinese medicine

## Abstract

**Objective:**

To evaluate the comprehensive therapeutic value of platelet-rich plasma (PRP) combined with antibiotic-loaded bone cement, systemic metabolic management, and topical herbal preparation for diabetic foot ulcer (DFU) with tophi.

**Methods:**

A 55-year-old male with tophaceous DFU received multimodal treatment, including debridement, antibiotic-impregnated bone cement implantation, local autologous PRP injections, topical Xiang Lei Tang Zu Ointment, and systemic control of glucose and uric acid. Efficacy was assessed via wound area, laboratory tests, and imaging.

**Results:**

After 4 months, the ulcers were nearly healed with significant reduction in wound area, healthy granulation tissue, and complete re-epithelialization. Serum uric acid decreased from 564 μmol/L to normal range, alongside reduced inflammatory markers.

**Conclusion:**

This multimodal strategy integrating local anti-infection, bioactive repair, topical agents, and systemic regulation effectively controlled infection, promoted tissue regeneration, and improved the metabolic microenvironment, offering a promising integrative model for refractory tophaceous DFU.

## 1 Introduction

Diabetic foot ulcer (DFU) is a common and severe complication of diabetes mellitus, characterized by high recurrence rates, high disability rates, and prolonged treatment duration. Epidemiological studies indicate a global DFU prevalence of approximately 5-10%, with an annual incidence of 1-4% (1). In China, DFU is a leading cause of hospitalization among diabetic patients, with an amputation rate as high as 22.4% (2). The pathophysiology of DFU is complex, involving multiple factors such as neuropathy, ischemia, infection, and metabolic disturbances, posing significant challenges for clinical management (3).

Current first-line conventional treatments for DFU include glycemic control, standard wound care—such as infection management, debridement, dressing changes, and topical agents-and revascularization for ischemic peripheral arterial disease (3). In recent years, biological therapies and tissue engineering have provided new directions for DFU treatment. Platelet-rich plasma (PRP) is an autologous biological preparation that is rich in various growth factors, including platelet-derived growth factor (PDGF), vascular endothelial growth factor (VEGF), and transforming growth factor-β (TGF-β). It has shown potential in promoting angiogenesis, cell proliferation, and tissue remodeling (4, 5). However, in complex DFU cases complicated by deep-seated infections (e.g., osteomyelitis) or concurrent metabolic disorders like gout, the efficacy of PRP treatment alone is often limited.

This article details the case of a patient with DFU and tophi who achieved wound healing through the sequential application of antibiotic-loaded bone cement, autologous PRP, topical herbal ointment, and strict management of blood glucose and uric acid levels. The purpose of this case report is to illustrate an integrated therapeutic strategy for such complex conditions. It also discusses the potential clinical application value and mechanisms of this approach, thereby providing a reference for clinical practice.

A 55-year-old male with BMI 27.8 kg/m^2^ was transferred to our hospital on July 4, 2025, because he had ruptured and infected tophi at the first metatarsophalangeal joints of both feet for more than two months. The patient had a 10-year history of type 2 diabetes and hyperuricemia, with long-term suboptimal control of both blood glucose and uric acid. His history was significant for poor adherence to prescribed oral hypoglycemic agents (metformin and gliclazide) and dietary recommendations. His most recent HbA1c prior to admission was 7.9% (normal range: 4.5-6.5%). There was no significant family history of diabetes or gout. Chronic complications included diabetic kidney disease. On admission, serum creatinine was 265 μmol/L, with an estimated glomerular filtration rate (eGFR) of 24 mL/min/1.73m² (CKD-EPI formula), and urine albumin-to-creatinine ratio (UACR) was 350 mg/g. According to the KDIGO 2024 guidelines (1), this corresponds to stage G4A3 (eGFR 15–29 mL/min/1.73m² with severely increased albuminuria). There was no documented retinopathy or macrovascular complications. A screening for peripheral arterial disease (PAD) using the ankle-brachial index (ABI) was performed on admission, yielding values of 1.08 on the right and 1.05 on the left, which were within the normal range and did not indicate significant PAD. The patient was a former smoker with a 20-pack-year history, having quit five years prior. His baseline lipid profile showed total cholesterol of 2.27 mmol/L (normal range: <5.2 mmol/L), triglycerides of 0.95 mmol/L (normal range: <1.81 mmol/L), LDL-C of 1.16 mmol/L (normal range: ≤3.4 mmol/L), and HDL-C of 0.74 mmol/L (normal range: ≥0/9 mmol/L). He was not on any lipid-lowering therapy at admission.

In May 2025, he noticed lumps about the size of soybeans at the first metatarsophalangeal joints of both feet. The lumps progressively enlarged, but without pain, skin redness, or ulceration, so the patient did not seek medical attention. In early June 2025, the tophi on the right first metatarsophalangeal joint spontaneously ruptured, presenting with redness, swelling, heat, pain, and fever. The wound did not heal despite antibiotic treatment and drainage at another hospital.

Informed consent for the publication of this case report and associated images was obtained from the patient.

## 2 Case presentation

### 2.1 Initial assessment and diagnosis upon transfer

**Figure 1:**
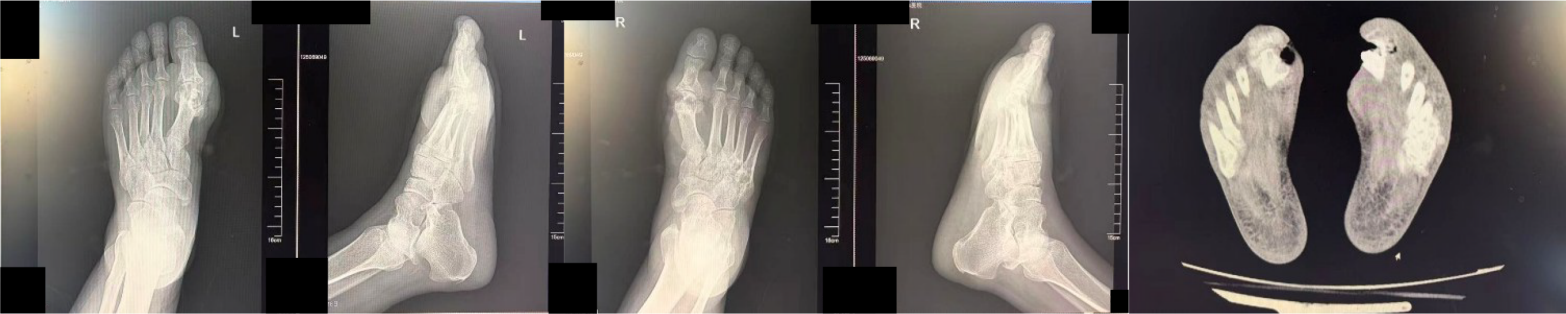
X-ray images of the patient’s bilateral feet.

**Figure 2:**
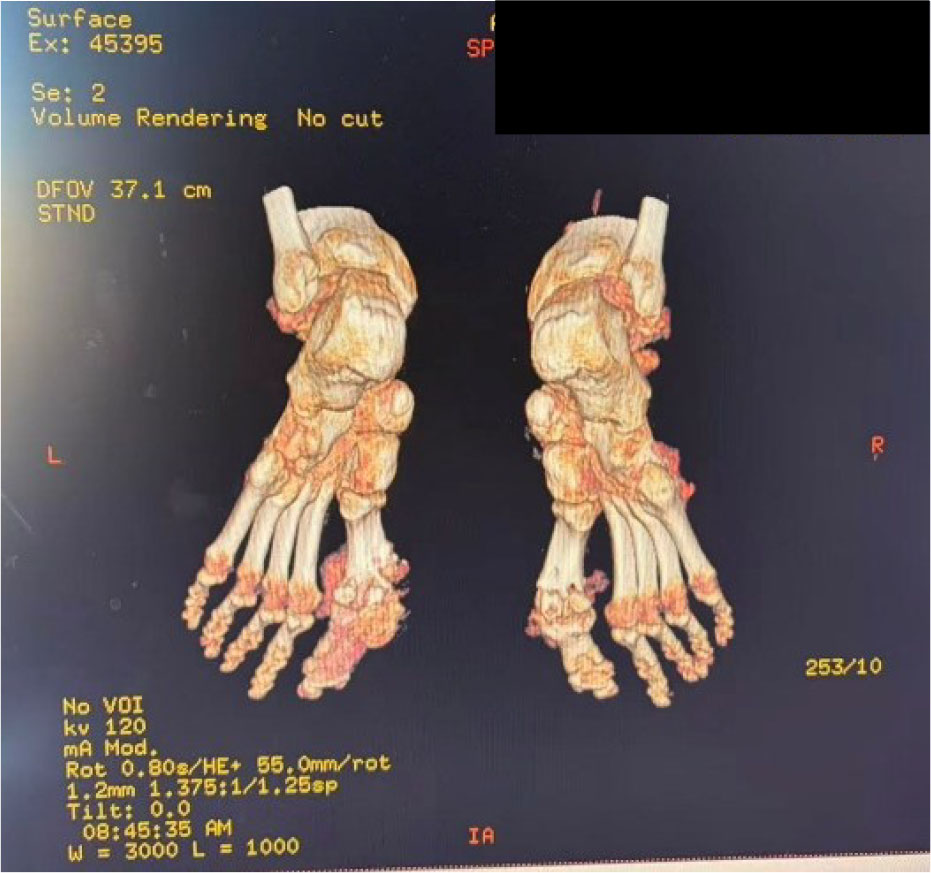
CT scans of the patient’s bilateral feet.

Examinations at the external hospital on June 20, 2025, showed elevated levels of high-sensitivity C-reactive protein (hs-CRP) at 273.7 mg/L (normal range: 0–5 mg/L) and serum uric acid at 564 μmol/L (normal range for males: 90-430 μmol/L). Renal function assessment upon transfer (as detailed in the case presentation) confirmed diabetic kidney disease stage G4A3 according to the KDIGO 2024 guidelines (1). Imaging studies (X-ray and CT) revealed bone destruction at the first metatarsophalangeal joints of both feet, accompanied by multiple high-density soft tissue nodules, consistent with diabetic foot osteomyelitis and tophus formation (**Figures 1**, **2**). The diagnosis was confirmed as: Type 2 diabetic foot ulcer (Wagner grade 3) with osteomyelitis, gout, and diabetic kidney disease stage G4A3.

### 2.2 Phase one: infection control and wound bed preparation (July 2025)

**Figure 3:**
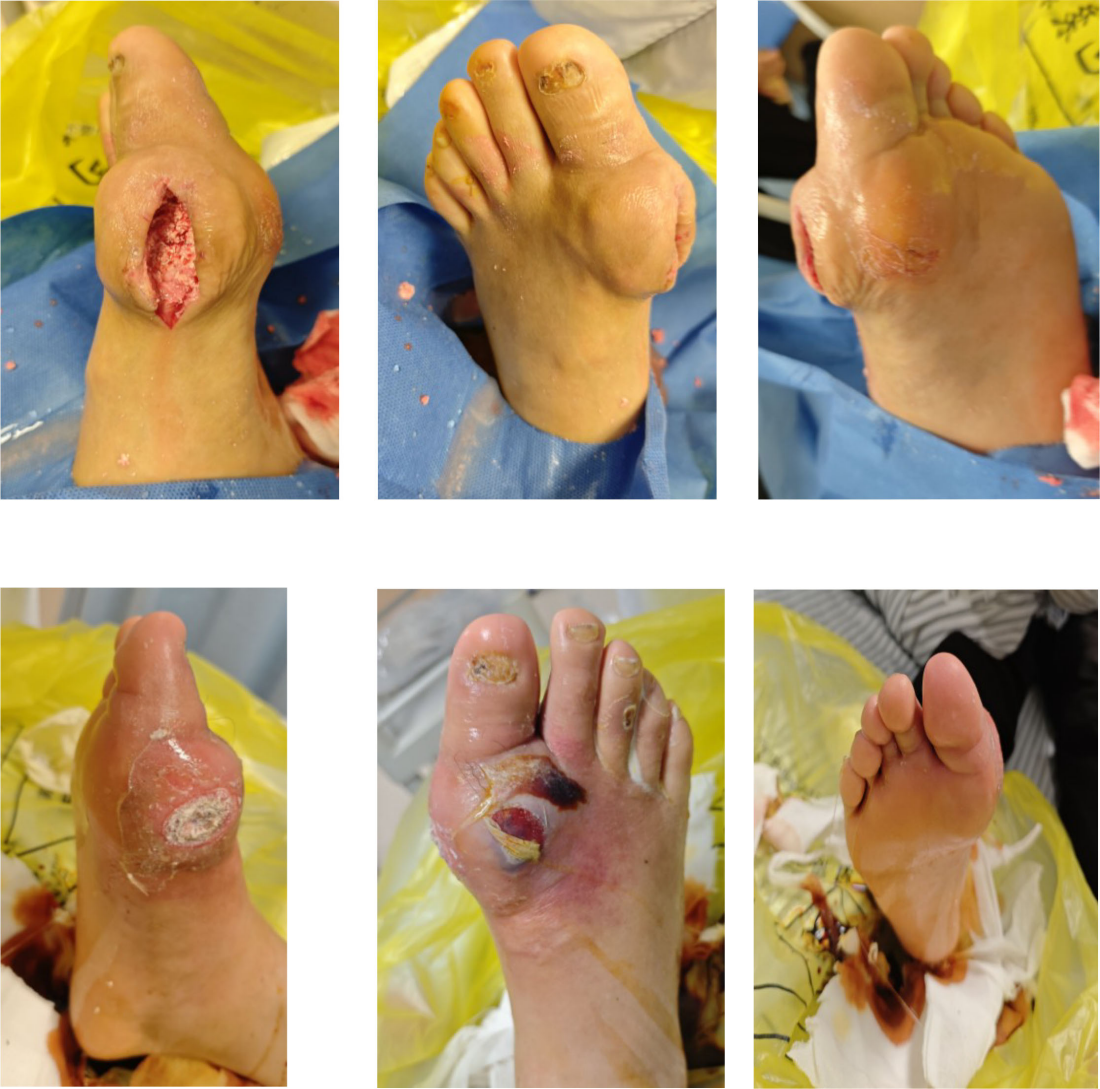
Preoperative wound dressing of the bilateral feet.

**Figure 4:**
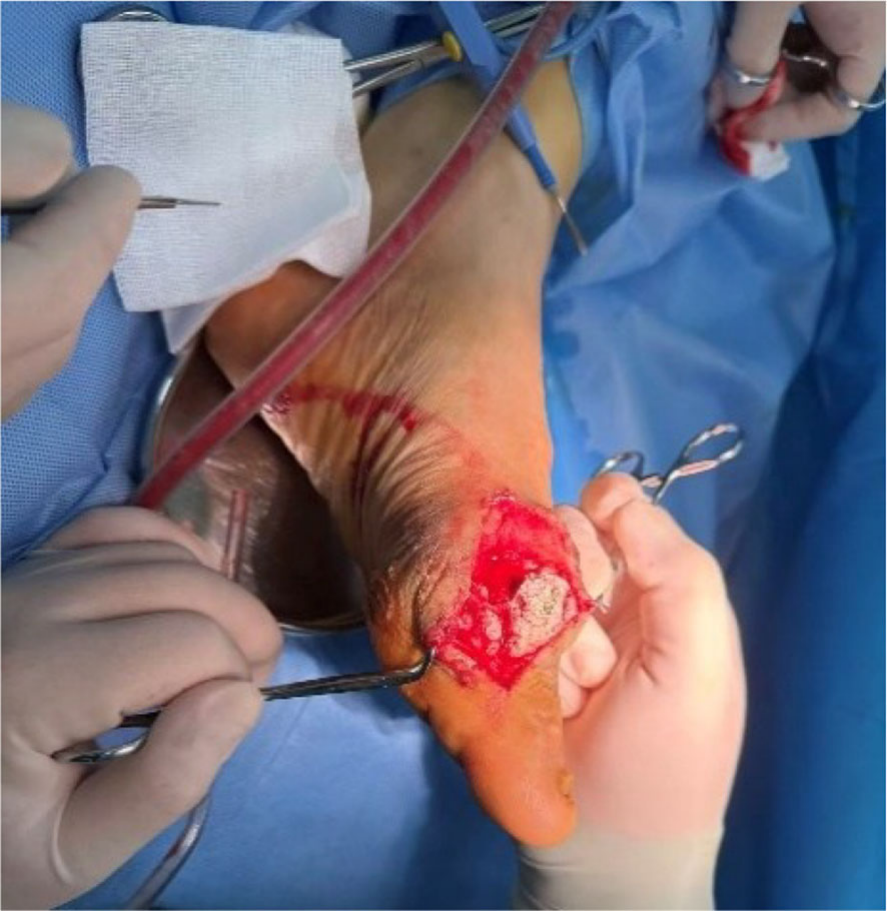
Intraoperative implantation of antibiotic-impregnated bone cement in the left foot.

**Figure 5:**
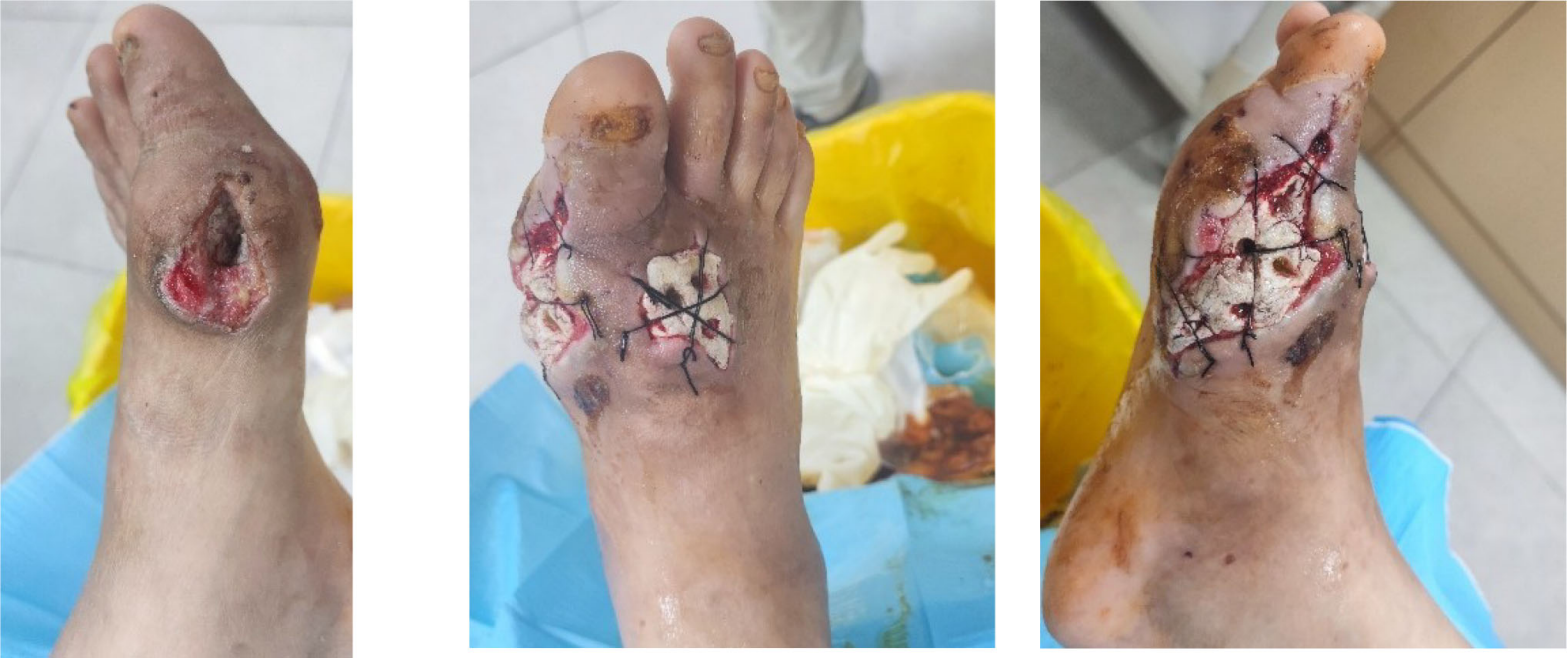
Bilateral feet wounds at initial presentation to our hospital.

The patient underwent debridement of both feet, removal of tophi, repair of chronic ulcers, and implantation of antibiotic-impregnated bone cement (containing vancomycin and gentamicin) (AIBC) on July 1 and July 11, 2025. Preoperative wound dressing is shown in **Figure 3**, and intraoperative implantation of bone cement is shown in **Figure 4**. Upon transfer to our hospital, a deep ulcer measuring approximately 4×4 cm with extensive tophi exposure persisted on the left foot (**Figure 5**). Laboratory tests indicated ongoing infection (hs-CRP 83.5 mg/L, erythrocyte sedimentation rate [ESR] 140 mm/1h (normal range: 0–15 mm/1h)] and acute kidney injury on the background of pre-existing diabetic kidney disease. The treatment regimen included: intensive insulin therapy for glycemic control; piperacillin-tazobactam for infection; febuxostat combined with sodium bicarbonate for urate-lowering and urine alkalinization; short-term corticosteroids for acute gout flare management; and renal protective therapy. Atorvastatin 20 mg/day was initiated for lipid management.

### 2.3 Phase two: initiation of regenerative repair (mid-August to early September 2025)

**Figure 6:**
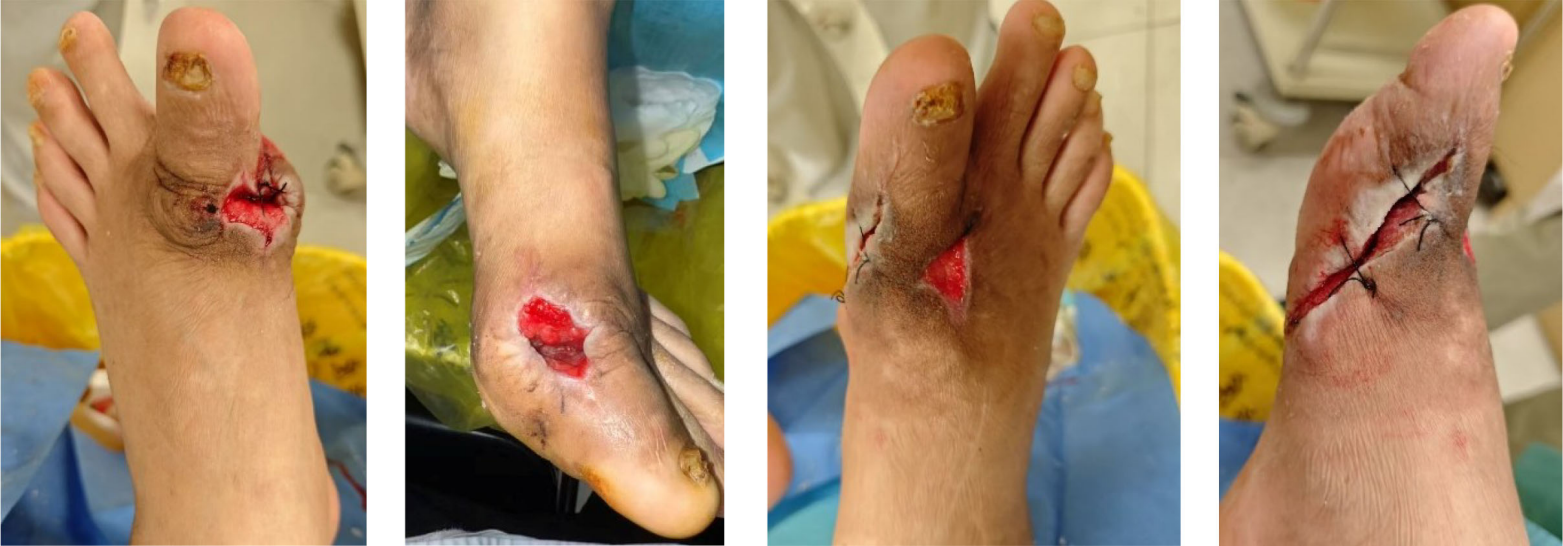
Bilateral feet after bone cement removal and repair.

**Figure 7:**
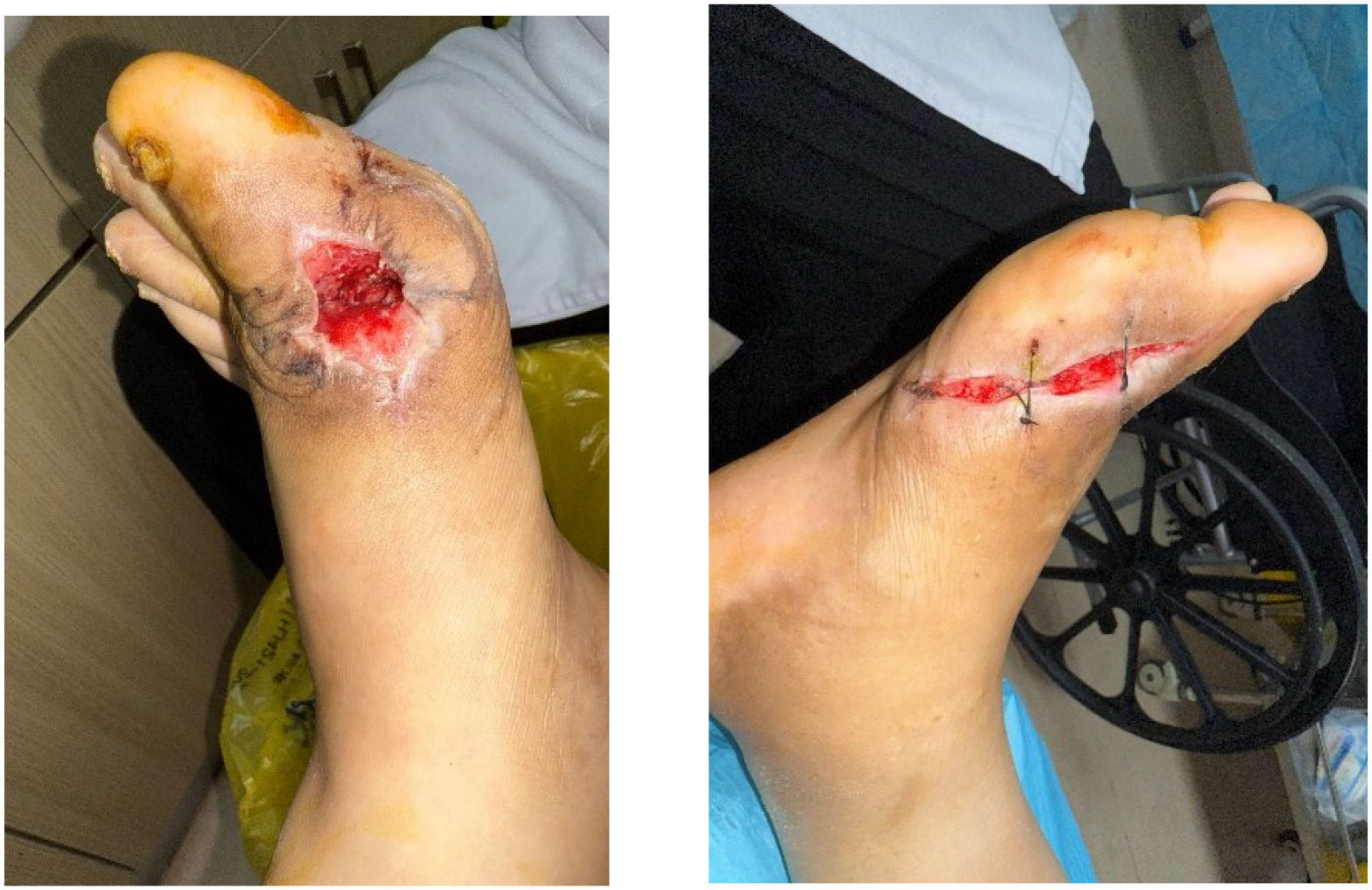
First session of PRP therapy.

**Figure 8:**
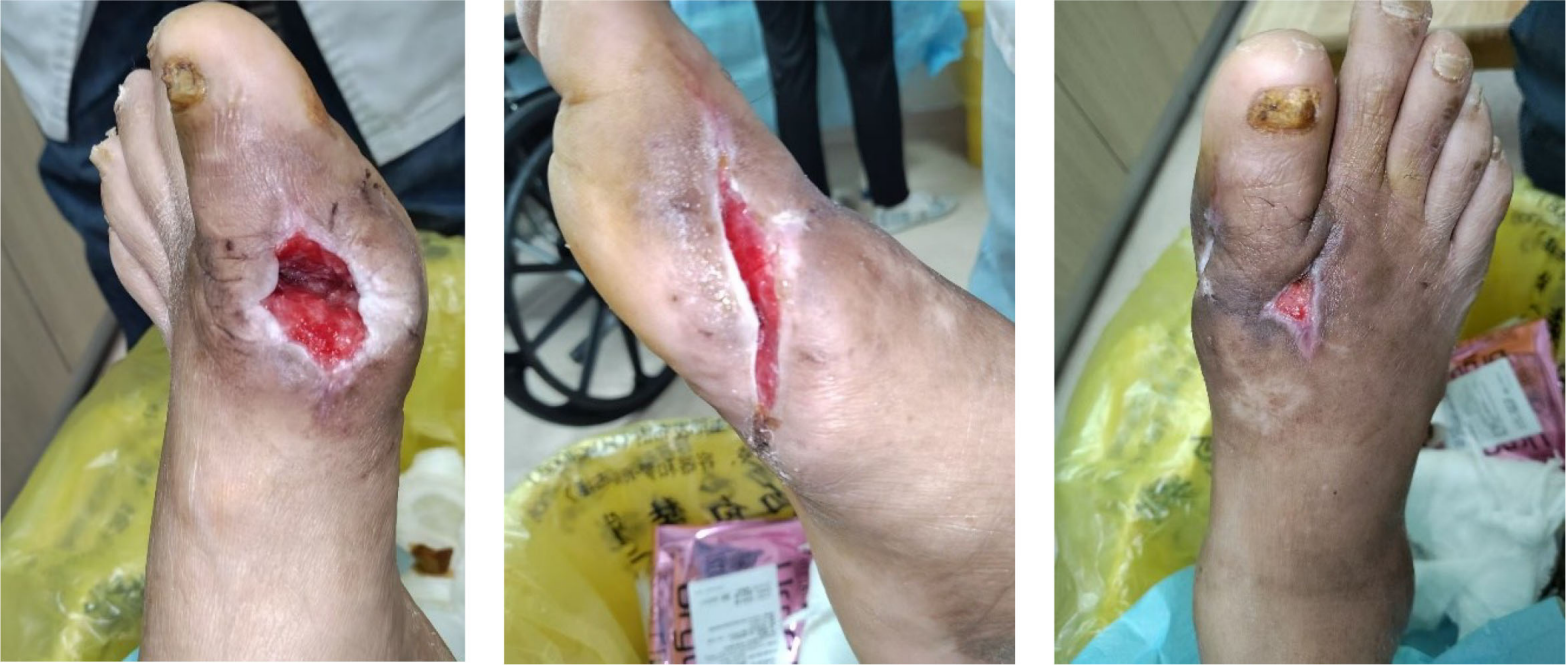
Second session of PRP therapy.

**Figure 9:**
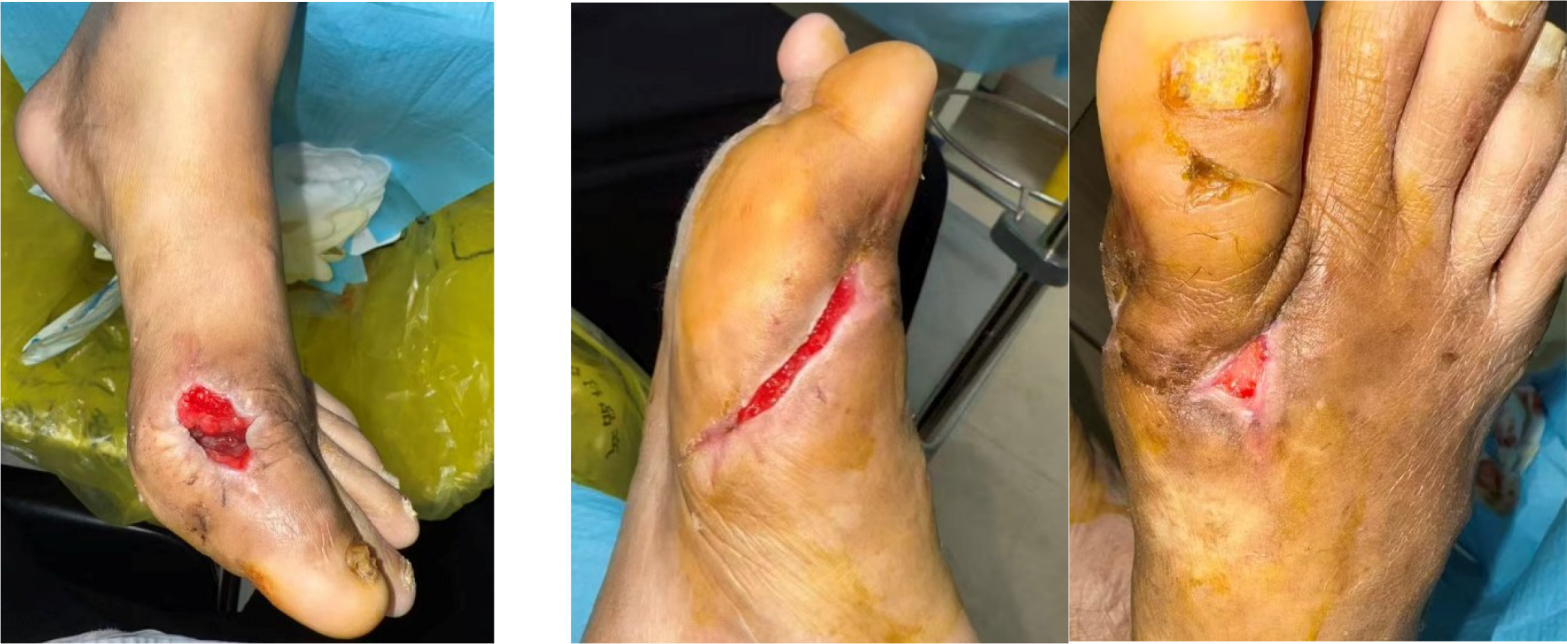
Third session of PRP therapy.

After removing the bone cement in mid-August, chronic ulcers measuring approximately 3×2 cm on the left foot and 1×5 cm on the right foot persisted, with poor granulation tissue growth (**Figure 6**). Serum uric acid rebounded to 551 μmol/L, higher than baseline levels. To promote healing, three sessions of local autologous PRP injection were administered on August 21, August 28, and September 4, 2025. PRP was prepared using a double-centrifugation method and, after activation, was evenly sprayed onto the wound bed (**Figures 7**–**9**). During this period, the patient maintained strict non-weight-bearing status and received febuxostat (40 mg/day) to stabilize uric acid levels.

### 2.4 Phase three: wound consolidation and follow-up (September to October 2025)

**Figure 10:**
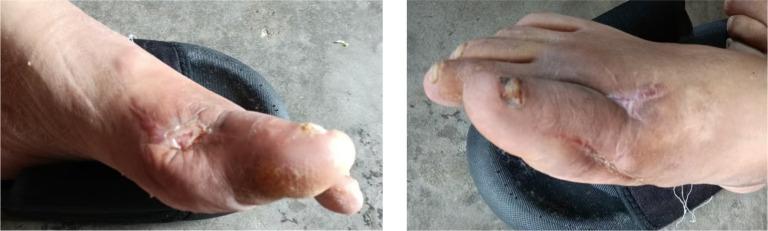
Three weeks after application of “Xiang Lei Tang Zu ointment”.

**Figure 11:**
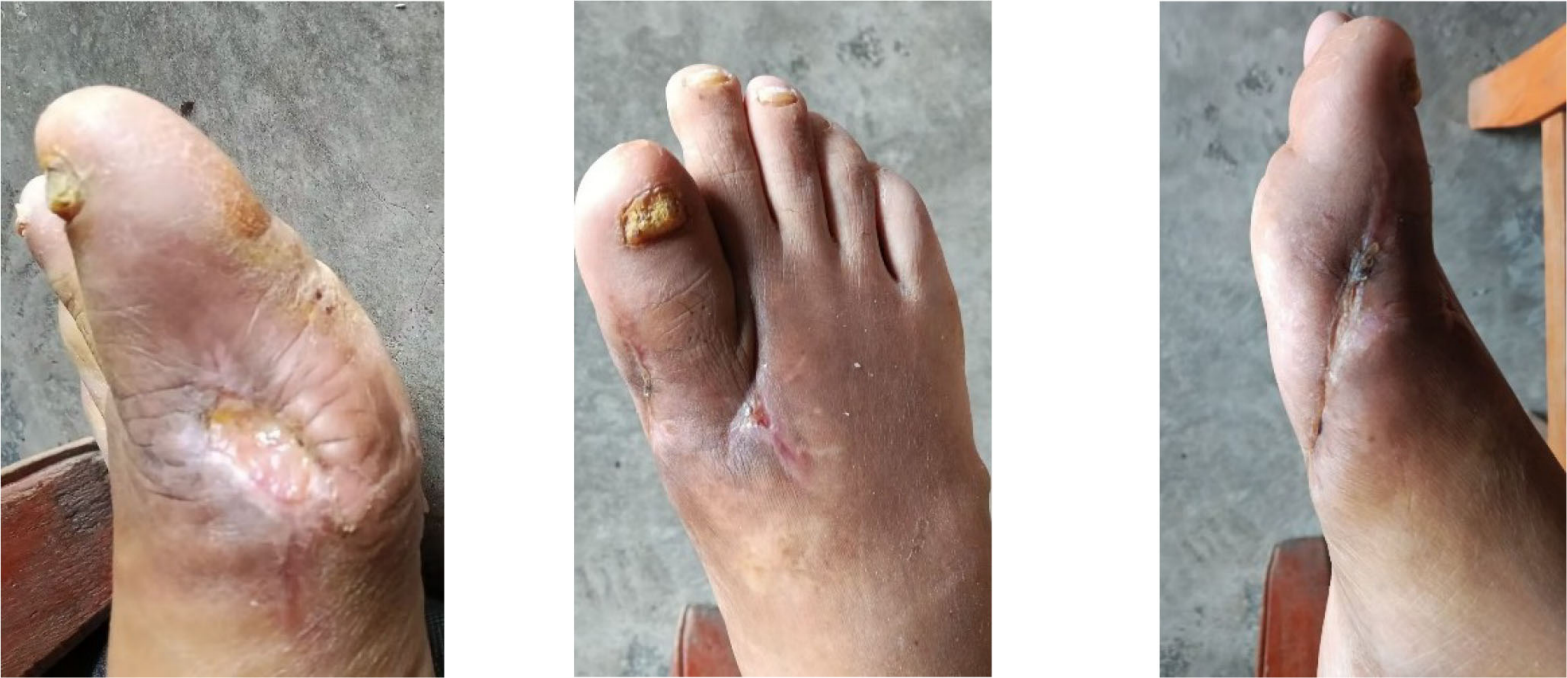
Initial application of “Xiang Lei Tang Zu ointment”.

**Figure 12:**
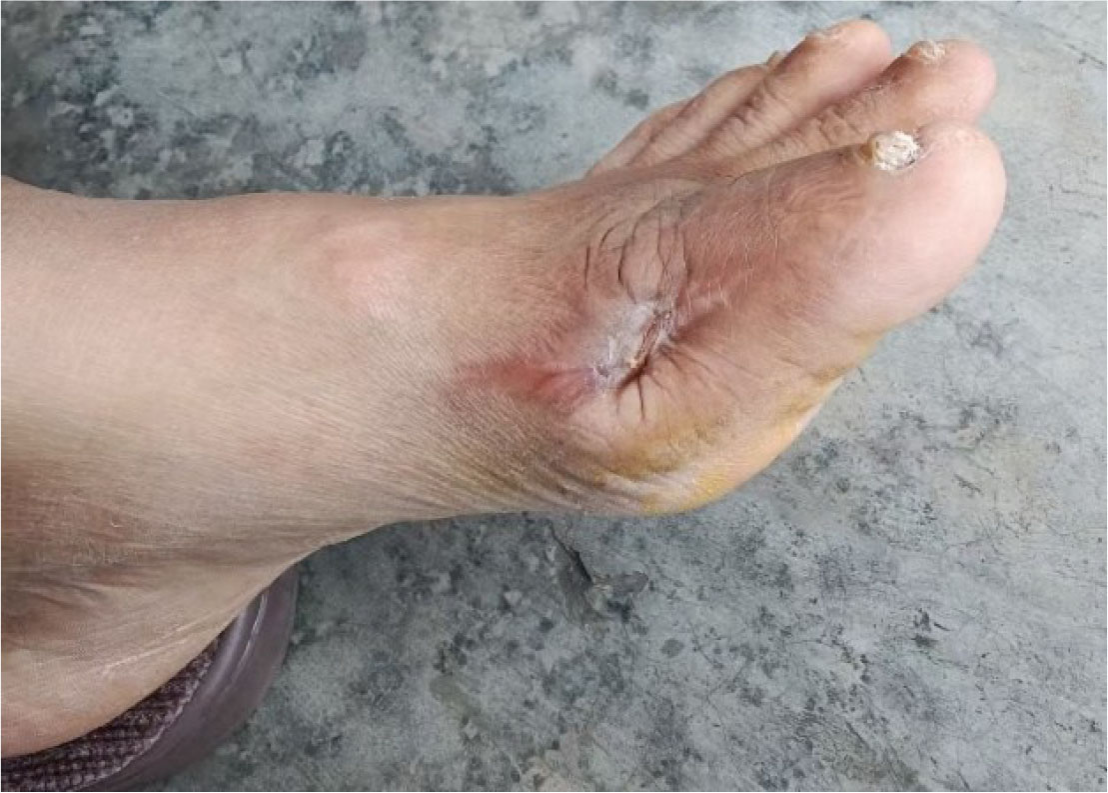
Near-complete wound healing.

After PRP treatment, wound sizes reduced significantly with robust, red granulation tissue. The patient was discharged on September 5, 2025, and then applied Xiang Lei Tang Zu Ointment three times weekly as maintenance therapy (**Figure 10**). The initial application of the ointment is shown in **Figure 11**. At the follow-up on October 21, 2025, the ulcers on both feet had essentially healed (**Figure 12**). Throughout the treatment course, serum uric acid levels were effectively controlled and maintained within the normal range, with a final follow-up value of 360 μmol/L. HbA1c impressed to 7.0%, reflecting enhanced glycemic control achieved through the intensive insulin regimen. Renal function also showed improvement, with serum creatinine decreasing to 145 μmol/L, eGFR increasing to 52 mL/min/1.73m², and UACR reducing to 120 mg/g, consistent with stabilization of the patient’s overall metabolic status. Overall systemic metabolic stability was achieved.

## 3 Discussion

### 3.1 DFU and gout/hyperuricemia: a vicious cycle of mutual aggravation

This case clearly illustrates the complexity of foot ulcers in the comorbidity of diabetes and gout. Both conditions are metabolic diseases that share insulin resistance as their common pathophysiological core (6). The hyperglycemic environment leads to advanced glycation end-product accumulation, oxidative stress, and neurovascular complications, which impede the normal wound healing process. Concurrently, hyperuricemia causes direct mechanical tissue damage and inflammation through urate crystal deposition (tophi). In addition, soluble urate can activate the NLRP3 inflammasome, promoting the release of pro-inflammatory cytokines such as interleukin-1β (IL-1β), thereby exacerbating local and systemic chronic inflammation (7, 8). This persistent inflammatory milieu severely disrupts the normal transition between the inflammatory, proliferative, and remodeling phases of wound healing. Studies indicate that serum uric acid levels in DFU patients are significantly higher than in diabetic patients without ulcers and are positively correlated with the severity of lower extremity vascular disease (9). In this case, long-term uncontrolled hyperuricemia and tophi formation were undoubtedly significant contributing factors to the protracted, recurrent nature of the DFU. Therefore, managing serum uric acid as a core goal, which is equally important to glycemic control, is key to breaking this vicious cycle (6).

### 3.2 AIBC: a critical barrier for local infection control

Furthermore, DFU complicated by osteomyelitis presents a major therapeutic challenge. Systemic antibiotics often fail to achieve effective concentrations in infected bone tissue compromised by ischemia and biofilm formation. AIBC provides an ideal local drug delivery system. For this patient, vancomycin (1000 mg) targeting Gram-positive cocci and gentamicin (160 mg) targeting Gram-negative bacilli were mixed into 25 g of bone cement. Incorporating high-dose antibiotics during cement polymerization allows the cement to release drugs over several weeks post-implantation, achieving local concentrations tens to hundreds of times higher than systemic administration. This effectively penetrates biofilms and eradicates pathogens (10). Furthermore, the bone cement acts as a temporary filler, providing a physical barrier against external contamination, reducing tissue exudate, and serving as a scaffold for granulation tissue growth. A systematic review (10) confirmed that AIBC significantly shortens healing time, reduces the frequency of debridement in infected DFU, and does not increase adverse event risks. In this case, the two-stage implantation of AIBC effectively controlled deep-seated infection, creating a “clean” wound bed foundation for subsequent regenerative therapy.

### 3.3 PRP: bridging the gap from “healing stagnation” to “regenerative activation”

Following infection control, wounds often enter a state of “healing stagnation”. In this phase, there are insufficient growth signals to drive cell proliferation and tissue reconstruction. PRP is a plasma preparation enriched with platelets. It can be classified into autologous PRP (Au-PRP) and allogeneic PRP (Al-PRP) depending on whether the blood source is from the same individual or a donor. Due to its autologous nature, Au-PRP is less prone to immune rejection, making it the predominant form used in clinical settings. The mechanism of action of PRP is multifaceted: (1) Providing high concentrations of growth factors. Activated PRP releases multiple growth factors, including transforming growth factor-beta (TGF-β), platelet-derived growth factor (PDGF), vascular endothelial growth factor (VEGF), keratinocyte growth factor (KGF), hepatocyte growth factor (HGF), fibroblast growth factor (FGF), epidermal growth factor (EGF), and insulin-like growth factor (IGF) (4). During tissue remodeling, growth factors such as PDGF, KGF, and TGF-β stimulate fibroblast differentiation into myofibroblasts. This process accelerates collagen matrix contraction. At the same time, factors like EGF, IGF, KGF, and HGF promote epithelial cell division and proliferation, which accelerates wound contraction and re-epithelialization (5, 11); (2)Constructing a fibrin scaffold: Fibrinogen in Au-PRP forms a three-dimensional network upon activation, aiding not only hemostasis but also providing a provisional matrix for cell migration, colonization, and tissue remodeling (12); (3)Modulating the inflammatory and immune microenvironment: PRP modulates macrophage polarization from the pro-inflammatory M1 phenotype to the pro-repair M2 phenotype and balances matrix metalloproteinases (MMPs) with their inhibitors (TIMPs). This reduces excessive proteolytic damage and protects the extracellular matrix (13); (4)Possessing inherent antimicrobial activity: Activated PRP contains various antimicrobial proteins that can inhibit Staphylococcus aureus, Staphylococcus epidermidis, Escherichia coli, Klebsiella pneumoniae, and methicillin-resistant S. aureus(MRSA), and may act synergistically with antimicrobial agents (14). Several meta-analyses have confirmed that PRP significantly improves DFU healing rates and reduces healing time (15, 16). In this case, three sessions of local PRP injection after bone cement removal and basic infection control successfully activated the regenerative program, leading to rapid, dense granulation tissue growth, which laid a solid foundation for final epithelialization.

### 3.4 Xiang Lei Tang Zu ointment: integrating traditional and western medicine to consolidate repair outcomes

After PRP therapy initiates the repair process, it is crucial to maintain and consolidate the repair effect. Xiang Lei Tang Zu Ointment, a compound topical herbal preparation, exerts complementary effects through its two main active components: Plectranthus amboinicus extract (PA-F4), which possesses potent anti-inflammatory properties by inhibiting NLRP3 inflammasome activation and the NF-κB pathway to reduce the release of pro-inflammatory cytokines such as IL-1β and TNF-α, thereby controlling chronic wound inflammation (8, 17); and Jixuecao (S1), which promotes fibroblast proliferation, collagen synthesis, and angiogenesis (18). This combination of anti-inflammatory and pro-repair mechanisms aligns well with the pathological requirements during the late stages of chronic wound healing. A phase III randomized controlled trial showed that treatment with Xiang Lei Tang Zu Ointment for 16 weeks significantly increased the complete healing rate of DFU to 60.7%, compared to 35.1% in the control group (17). Subgroup analysis of DFU-related risk factors showed superior healing-promoting ability for ulcers including Wagner grade 2 (p < 0.0001), plantar ulcers (p = 0.0016), ulcer area ≥5 cm² (p = 0.0122), ulcer duration ≥3 months (p = 0.0043), patients with HbA1c ≥9% (p = 0.0285), and patients with BMI ≥25 kg/m² (p = 0.0005) (18). Furthermore, emerging evidence suggests that its bioactive components exert multifaceted therapeutic effects by modulating key signaling pathways related to oxidative stress, macrophage polarization, and extracellular matrix remodeling (17). In this case, continued use of Xiang Lei Tang Zu Ointment after PRP treatment effectively promoted epithelial migration and ultimately achieved wound closure. This highlights the advantages of integrating traditional Chinese and Western medicine in treating chronic wounds.

### 3.5 Construction and clinical value of a multimodal sequential treatment strategy

The success of this case is not due to a single therapy but to a logical, phase-defined multimodal sequential treatment strategy. Phase One (Debridement and Anti-infection) focused on surgical debridement and AIBC. The goal was to thoroughly remove necrotic tissue and tophi, control local infection, and create a “clean” wound environment for healing. Phase Two (Bioactive Repair) follows infection control, with Au-PRP serving as the core intervention. By delivering high concentrations of growth factors and providing a biological scaffold for repair, this phase aimed to activate and accelerate tissue regeneration, overcoming the “healing stagnation period.” Phase Three (Repair Consolidation and Epithelialization) centers on topical healing-promoting agents, specially Xiang Lei Tang Zu Ointment. This phase aims to maintain an anti-inflammatory, pro-repair local microenvironment, facilitating final epithelial coverage. Systemic management is throughout all phases. This includes meticulous glycemic control, consistent urate-lowering therapy (febuxostat), inflammation control during acute phases with short-term corticosteroids, and renal protection. These measures provide a stable systemic metabolic environment that supports local wound healing.

This integrated sequential strategy from anti-infection to pro-regeneration to consolidation of healing - systematically addresses the multiple barriers encountered in treating complex DFU with tophi, including infection, growth factor deficiency, chronic inflammation, and metabolic dysregulation. This approach serves as a valuable clinical model. It emphasizes that treatment should not merely combine various methods indiscriminately. Instead, it requires a stepwise, focused, and dynamic intervention tailored to the wound’s pathophysiological characteristics at each stage.

However, as a single case report, this study has inherent limitations. The multimodal nature of the intervention, with therapies applied sequentially, makes it impossible to isolate the individual efficacy of each component (e.g., PRP vs. AIBC vs. topical ointment). The observed clinical success is likely synergistic, and the relative contribution of each therapy cannot be definitively determined. Therefore, the findings should be interpreted as demonstrating the feasibility and potential of this integrated approach, which requires validation through larger, controlled studies.

## 4 Conclusion

Managing diabetic foot ulcers complicated by tophi is a clinical challenge. It requires multidisciplinary collaboration and individualized comprehensive strategies. This strategy embodies the integrative medical principles of “balancing local and systemic approaches,” “combining anti-infection and pro-repair measures,” and “combining modern technology with traditional medicine.” In this case, strict management of cardiovascular risk factors, including the initiation of statin therapy and intensive glycemic control, alongside the multimodal wound care, contributed to the overall favorable outcome. Although this single case report achieved favorable outcomes, future rigorously designed, large-sample randomized controlled trials are necessary to further validate the efficacy of this sequential protocol, optimize treatment parameters (e.g., PRP preparation standards, injection frequency and dose, antibiotic combinations in bone cement), and investigate its molecular mechanisms in depth. This will provide evidence-based, efficient treatment options for a broader patient population.

## Data Availability

The original contributions presented in the study are included in the article/supplementary material. Further inquiries can be directed to the corresponding author.
