# Correction: Platelet-rich plasma combined with multimodal therapy for diabetic foot ulcer with tophi: a case report

**DOI:** 10.3389/fcdhc.2026.1863254

**Published:** 2026-05-05

**Authors:** Xinjie Duan, Xinjuan Sun, Yang Liu, Maoyuan Chen, Li Dong

**Affiliations:** 1 Department of Endocrinology, Nanjing Red Cross Hospital, Nanjing, China; 2 Department of Endocrinology, The Air Force Hospital from Eastern Theater of PLA, Nanjing, China

**Keywords:** antibiotic-impregnated bone cement, diabetic foot ulcer, platelet-rich plasma, tophi, traditional Chinese medicine

Author Xinjie Duan was erroneously assigned to affiliation “Department of Endocrinology, The Air Force Hospital from Eastern Theater of PLA, Nanjing, China.” This affiliation has now been removed for author Xinjie Duan.

Author Xinjuan Sun was erroneously assigned to affiliation “Department of Endocrinology, Nanjing Red Cross Hospital, Nanjing, China.” This affiliation has now been removed for author Xinjuan Sun.

Author Yang Liu was erroneously assigned to affiliation “Department of Endocrinology, The Air Force Hospital from Eastern Theater of PLA, Nanjing, China.” This affiliation has now been removed for author Yang Liu.

Author Maoyuan Chen was erroneously assigned to affiliation “Department of Endocrinology, The Air Force Hospital from Eastern Theater of PLA, Nanjing, China.” This affiliation has now been removed for author Maoyuan Chen.

Author Li Dong was erroneously assigned to affiliation “Department of Endocrinology, The Air Force Hospital from Eastern Theater of PLA, Nanjing, China.” This affiliation has now been removed for author Li Dong.

The original version of this article has been updated.

